# Bilateral Gonadal Dysgerminoma in a Phenotypic Female With 46,XY Disorder of Sexual Development: A Case Report

**DOI:** 10.7759/cureus.38149

**Published:** 2023-04-26

**Authors:** Ricardo Pasquini Neto, Maria Letícia Carnielli Tebet, Ohana Ivanski Dória de Vasconcelos, Mariana Faucz Munhoz da Cunha, Maria Cristina Figueroa Magalhães

**Affiliations:** 1 School of Medicine, Pontifícia Universidade Católica do Paraná, Curitiba, BRA; 2 School of Medicine, Faculdade Evangélica Mackenzie do Paraná, Curitiba, BRA; 3 Department of Pediatric Nephrology, Hospital Pequeno Príncipe, Curitiba, BRA; 4 Department of Clinical Oncology, Hospital Universitário Evangélico Mackenzie, Curitiba, BRA

**Keywords:** 46xy disorder of sex development, 46xy gonadal dysgenesis, primary and secondary amenorrhea, ovarian dysgerminoma, germ cell and embryonal neoplasms, sex determining region y, sex reversal syndrome, disorders of sex development

## Abstract

The 46,XY disorder of sexual development (DSD) is a rare congenital condition characterized by a 46,XY karyotype associated with complete or disturbed female gonadal development and a non-virilized phenotype. The presence of Y chromosome material in these patients' karyotypes increases the risk of germ cell tumor development. The present study reports a unique case of a 16-year-old phenotypically female patient presenting with primary amenorrhea, who was later diagnosed with 46,XY DSD. After bilateral salpingo-oophorectomy, the patient was diagnosed with stage IIIC dysgerminoma. The patient received four cycles of chemotherapy and showed a good response. The patient is currently alive and well, with no evidence of disease after the residual lymph node resection.

## Introduction

The 46,XY disorder of sexual development (DSD) is a mismatch between the gonadal sex and the chromosomal sex [1,2]. Despite having a 46,XY karyotype, patients exhibit a non-virilized phenotype, female external genitalia, Müllerian structures, and no sperm production [3]. When the gonads lack hormonal and reproductive potential, symptoms usually appear in adolescents with delayed puberty and primary amenorrhea [4]. This syndrome occurs in approximately one in 5,000 live births [5].

Currently, the exact molecular and genetic basis of 46,XY DSD remains unclear. Patients with Y chromosome material in their karyotype have an increased risk of developing germ cell tumors, particularly gonadoblastomas, and dysgerminomas [1,3,5].

The present study reports a unique case of bilateral dysgerminoma in a girl presenting with primary amenorrhea who was later diagnosed with 46,XY DSD. It describes the clinical diagnostic approach, surgical aspects, and oncologic treatment that resulted in a good response.

## Case presentation

A nine-year-old female patient presented to her annual primary care visit with a routine laboratory evaluation revealing elevated creatinine levels (11.8 mg/dL, normal range: 0.5-1 mg/dL) and urea levels (93 mg/dL, normal range: 8-20 mg/dL), and a decreased glomerular filtration rate (4.4 mL/min/1.73 m^2 ^according to the Schwartz formula, normal range over 90 mL/min/1.73 m^2^) [6,7]. The patient was asymptomatic and had no signs of proteinuria, altered albumin-creatinine ratio, or hematuria in a partial urine specimen. Given the abnormal laboratory findings, the patient was referred to a tertiary center for a comprehensive evaluation, including laboratory tests and imaging studies to determine the kidney disease etiology.

The patient was born via normal full-term delivery to unrelated parents after an uncomplicated pregnancy. Maternal use of sex hormones or radioactive substances was not reported. There was no family history of genetic diseases or known malignancies. The patient had a healthy childhood with appropriate neuropsychomotor and growth development.

Upon admission to the pediatric nephrology outpatient clinic, the patient was clinically stable, cooperative, asymptomatic, and had preserved micturition. The initial examination revealed stage I systemic arterial hypertension (systolic blood pressure of 120 mmHg - percentile 95 + 7 mmHg; diastolic blood pressure of 80 mmHg - percentile 95 + 5 mmHg) and stature below the familial range-25th percentile of the World Health Organization height-for-age chart (girls) 5-19 years [8]. Pedal edema and peritoneal, pleural, and pericardial effusion were absent. Pre-micturition and post-micturition volumetric renal ultrasonography revealed kidneys with typical topography and reduced dimensions (right kidney - 5.0 x 2.5 x 3.5 cm; left kidney - 6.2 x 2.8 x 3.2 cm), pelvicalyceal dilatation (anteroposterior pelvic diameter of 2.3 cm), and thickened-walled bladder (0.7 cm). Considering a nephropathy of probable post-renal etiology and the absence of detectable obstruction, a urodynamic study was requested to assess the functionality and integrity of the urinary system. On the occasion, cystourethrography did not reveal vesicoureteral reflux or other alterations.

In the next week, a peritoneal dialysis (PD) catheter was placed with minimal access, and the patient began PD. The treatment lasted for five months until kidney transplantation which was well-tolerated. The renal allograft was donor-matched and immunosuppression, consisting of tacrolimus, mycophenolate mofetil, and corticosteroids, was performed to prevent rejection episodes.

In the following years, the patient was regularly followed up at the renal transplant service. At 16 years of age, she did not reach menarche, despite the presence of secondary sexual characteristics, including breast enlargement and pubic/axillary hair development. The patient was referred to the pediatric gynecology department for evaluation of primary amenorrhea.

A gynecological evaluation revealed a feminine appearance and voice without a beard or laryngeal prominence. The external genitalia was female without ambiguity or clitoral enlargement, and the hymen was intact. Secondary sexual characteristics were defined as M4 P4 according to the Tanner scale [9]. The patient reported thelarche at 12 years of age, and her mother’s menarche at 16 years of age. Her height was above the familial range of the 85th percentile on the World Health Organization’s height-for-age chart (girls) of 5-19 years [8]. She was overweight (body mass index of 26 kg/m^2^ -86.9th percentile on the World Health Organization’s body mass index-for-age chart (girls) of 5-19 years), with a waist-to-hip ratio of > 80%. The morphological evaluation revealed no body disproportion (arm span was proportional to the patient’s height) [8].

B-mode ultrasound showed a uterus (55.4 x 19.9 x 23.2 mm (13.6 cm^3^)) with a thin and homogeneous endometrium, fallopian tubes, and the upper part of the vagina. The right gonad measured 13.5 x 19.1 x 12.2 mm (1.6 cm^3^) and the left gonad was 15.4 x 11.9 x 10.2 mm (1 cm^3^); both were regular and homogeneous, with hyperechogenic images suggestive of follicular development. These findings are consistent with those of the prepubertal phase. Hand and wrist radiographs showed a bone age compatible with 15 years, according to the Greulich-Pyle atlas [10]. Continuous outpatient follow-up was done after the analysis of late menarche.

Six months after the initial evaluation of amenorrhea, the patient did not experience menarche. Serum sex hormone levels revealed hypergonadotropic hypogonadism. Thyroid-stimulating hormone and free T4 were within normal ranges. The patient’s total serum testosterone levels were slightly elevated (Table 1) [6]. As this was a case of primary amenorrhea of probable ovarian etiology, the G-banding karyotype revealed 46,XY in two samples collected on different days. No deletions were detected at the sY84, sY86, sY127, sY134, sY254, or sY255 loci in the sex-determining region Y protein/testis-determining factor gene (SRY/TDF). Due to financial difficulties and availability constraints in the Brazilian healthcare system, only SRY Sanger sequencing was performed. The patient was diagnosed with 46,XY DSD, and simple gonadal dysgenesis. The patient and her mother underwent counseling after diagnosis; however, as she was raised as a girl and was comfortable about the situation, she declined the gender conversion process.

**Table 1:** Primary amenorrhea laboratory evaluation

Laboratorial Parameters	Patient's Results	Reference Ranges
Follicle-Stimulating Hormone (IU/L)	97.8	5–20 (female, follicular/luteal phase); 30–50 (female, midcycle peak); > 35 (female, postmenopausal); 5–15 (male)
Luteinizing Hormone (IU/L)	69.1	5–22 (female, follicular/luteal phase); 30–250 (female, midcycle peak); > 30 (female, postmenopausal); 3–15 (male)
Estradiol (pg/mL)	<15	14–27 (female, day 1–10 of menstrual cycle); 14–54 (female, day 11–20 of menstrual cycle); 19–40 (female, day 21–30 of menstrual cycle); 10–30 (male)
Progesterone (ng/mL)	<0.1	< 1 (female, follicular phase); 3–30 (female, luteal phase)
Testosterone (total) (ng/dL)	80.1	20–75 (female); 300–1200 (male)
Thyroxine (T4) (ng/dL)	1.0	0,9–2,4
Thyroid-Stimulating Hormone (mIU/L)	2.7	0,5–5,0

**Figure 1:**
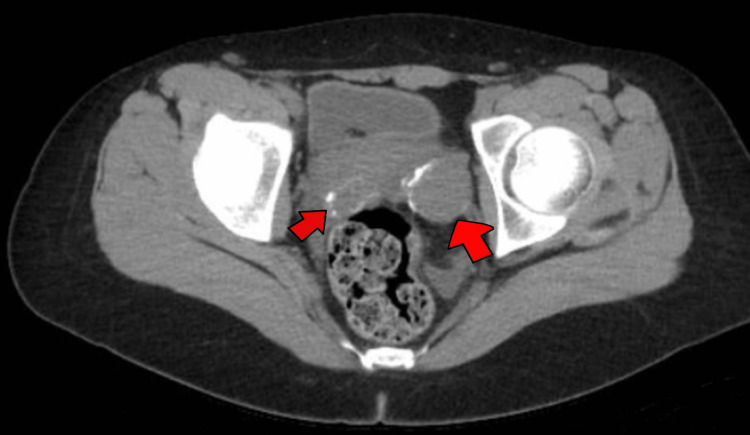
Computed tomography scan of the pelvis The exam revealed oval images with intermediate density and calcifications in bilateral adnexal region (arrows), measuring 30 mm on the right side and 28 mm on the left side. The findings were suggestive of primary gonadal tumoral lesions.

**Figure 2:**
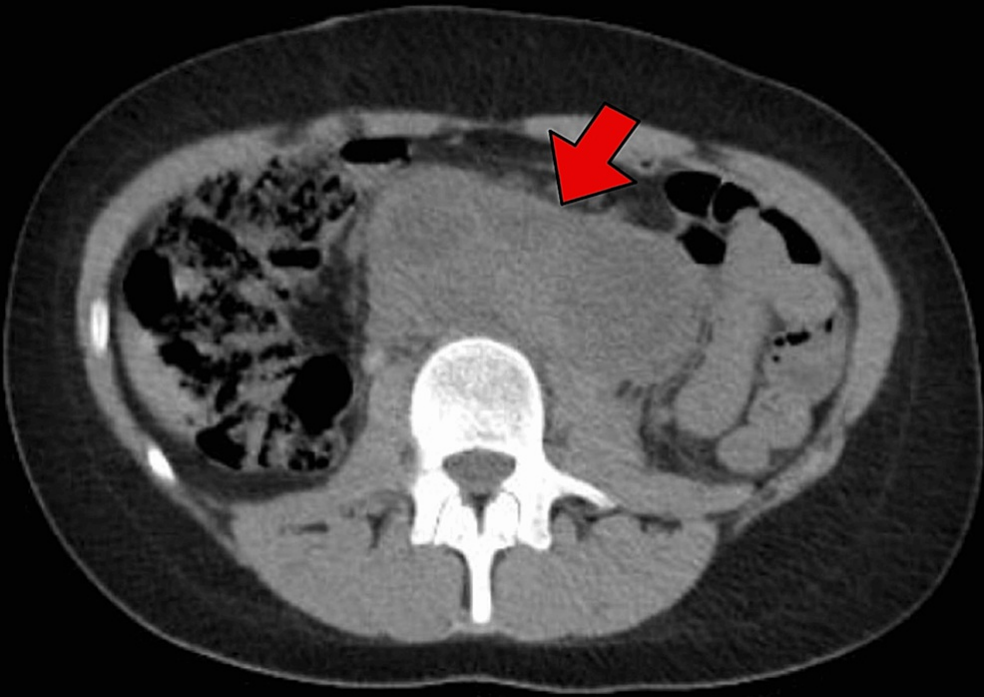
Computed tomography scan of the abdomen The exam revealed a retroperitoneal expansive lesion located in the epi/mesogastrium. The lesion was lobulated, predominantly hyperdense with intervening hypodense areas (arrow). It measured 12.0 x 9.8 x 4.7 cm and surrounded the aorta and the inferior vena cava (not shown in the image). The findings were suggestive of secondary retroperitoneal tumoral lesion.

While awaiting karyotyping and SRY/TDF sequencing, the patient presented to the pediatric emergency department with recurrent episodes of moderate-to-severe lower abdominal pain that awakened her while sleeping and was associated with unquantified weight loss. Physical examination revealed a distended abdomen, normal bowel sounds, diffuse tenderness upon deep palpation, and absence of lymphadenopathy. The recurrent abdominal pain was evaluated with a computed tomography (CT) scan that revealed primary tumor lesions in both ovaries (Figure 1) and secondary lesions in the retroperitoneal area (Figure 2). Notably, prophylactic gonadal resection could not be performed because the neoplasms were detected before the results of the genetic study were processed and gonadal dysgenesis was diagnosed.

The patient was referred to the pediatric oncology department for treatment planning due to the neoplastic diagnosis. The patient underwent bilateral salpingo-oophorectomy to resect the gonadal tumors and an incisional biopsy of the retroperitoneal mass owing to its unresectability. The anatomopathological analysis described the tumor as 6.5 cm (in the right gonad) and 4.5 cm (in the left gonad) in diameter at its largest axis, extending to the gonadal surface. Necrotic areas and lymphovascular infiltrates consisting of hyalinization and dystrophic calcification were noted in the adjacent tissues. There was no evidence of gonadal follicles or hormone production by the tumor. The gonadal histology revealed an ovarian stroma replaced by fibrous content. The findings suggested dysgerminomas in the dysgenetic gonads extending to the retroperitoneum, stage IIIC (T2b N1 M0), according to the American Joint Committee on Cancer [11]. There was no omental or peritoneal neoplastic involvement. Ascitic fluid cytology did not detect any malignant cells. With the exception of lactate dehydrogenase, which was elevated (618 IU/L), tumor markers were within the normal range (human chorionic gonadotropin beta less than 2.39 mlU/mL and alpha-fetoprotein less than 0.5 ng/mL).

The patient turned 18 years of age two days after the oncologic surgery and was transferred to the clinical oncology department to begin adjuvant chemotherapy. The treatment consisted of four cycles of etoposide 150 mg/m^2^/day (with a 25% dose reduction due to renal impairment) between D1 and D3, combined with carboplatin 600 mg/m^2^/day on D2, administered every 21-28 days according to possible neutropenia secondary to treatment. Bleomycin 10 mg/m^2^/week was administered starting on D3 of the first cycle and continued until the end of the fourth cycle (12 weeks) [12].

Based on the good response and tolerability of the first chemotherapy cycle, a switch to etoposide 120 mg/m^2^/day and cisplatin 20 mg/m^2^/day between days 1-5 in combination with the previously initiated bleomycin was proposed for the second cycle [11]. The third and fourth chemotherapy cycles were administered using the same regimen as the second, but cisplatin was administered only on days 1-4 and with the dose reduced by 25% owing to the risk of developing acute renal failure. 

The patient's response to chemotherapy was evaluated at the end of the fourth cycle. Lactate dehydrogenase levels normalized, and other tumor markers remained within the normal range. Retroperitoneal lymph node enlargement decreased; however, there was still detectable residual disease detected by an abdominal CT scan that was completely resected in a second minimally invasive surgery. The anatomopathological revealed exclusively necrotic tissue and it was decided to observe the patient with a physical exam and serum tumor markers every three months during the first year, every four months during the second year, every six months during the third, fourth, and fifth years, and annually after the fifth year. Abdominal CT should be taken every four months during the first year, every six months during the second year, annually during the third, fourth, and fifth years, and as clinically indicated after the fifth year [11]. Currently, the patient is stable, with quarterly follow-ups in the first year after completing the oncological treatment.

## Discussion

**Figure 3:**
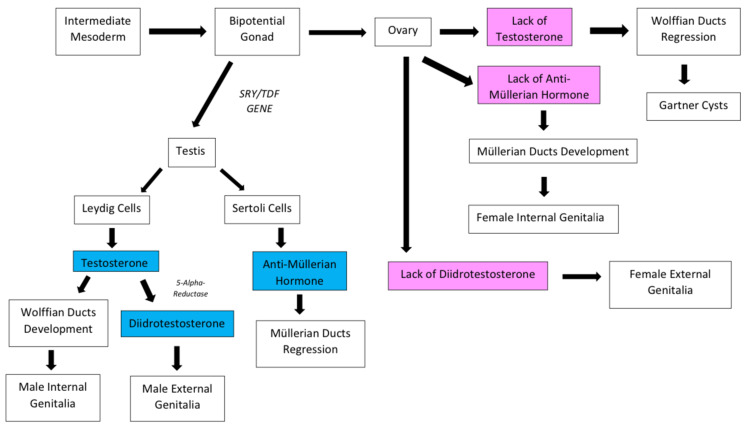
Development of the male and female reproductive systems SRY/TDF: sex-determining region Y protein/testis-determining factor gene Image credits - Ricardo Pasquini Neto, Maria Letícia Carnielli Tebet, Ohana Ivanski Dória de Vasconcelos, Mariana Faucz Munhoz da Cunha, and Maria Cristina Figueroa Magalhães

Genetic sex determination in mammals occurs during fertilization with karyotype formation, but reproductive system development begins only during embryogenesis, when the embryonic bipotential gonad commits to male or female differentiation [2]. The path followed by the primordial gonad depends on the presence of SRY/TDF on the short arm of the Y chromosome. By the eighth week of embryonic fertilization, the absence of SRY/TDF leads to gonadal differentiation into an ovary, with consequent Müllerian ducts development and Wolffian ducts regression (Figure 3) [2,13].

Proper regulation of the SRY/TDF gene is necessary for the proper development of the gonads and internal and external genitalia [1]. Mutations, deletions, or translocations may contribute to 46,XY DSD [3]. In this case, although the patient had the SRY/TDF gene without mutations in its major Y chromosome locus, she had no testes, but an ovarian cortex with normal female genitalia. This suggests that male gonadal development depends on genes other than SRY/TDF [3,14].

**Figure 4:**
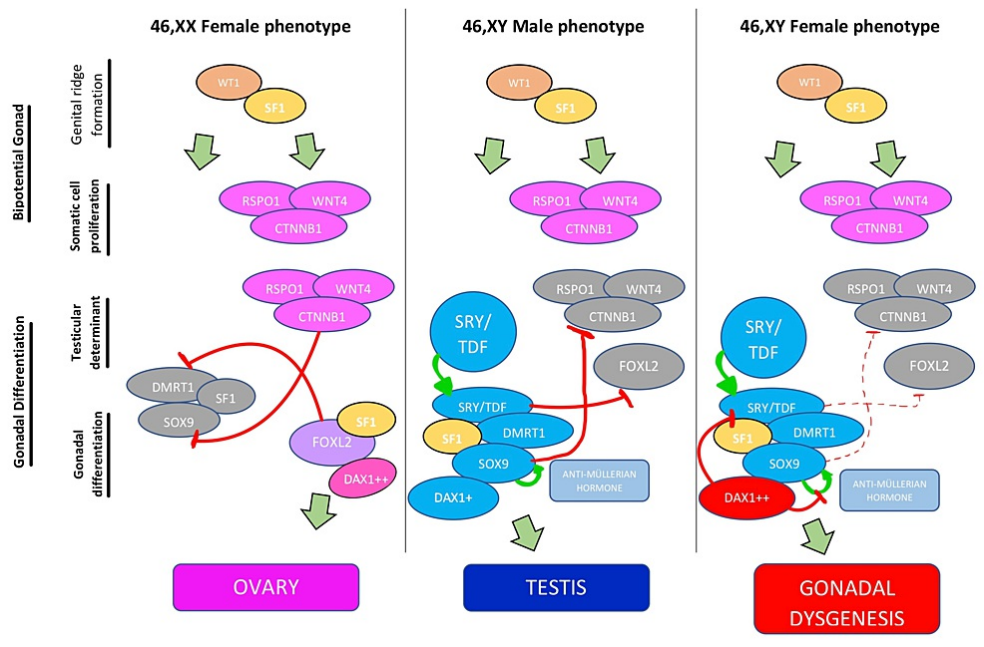
Genetic model of gonadal differentiation in female, male, and 46,XY disorder of sexual development with a duplication of the DAX1 gene WT1: Wilms tumor suppressor gene; SF1: steroidogenic factor-1; RSPO1: R-spondin-1 gene; WNT4: WNT family member 4 gene; CTNNB1: catenin beta-1 gene; DMRT1: double sex and mab-3-related transcription factor 1; SOX9: sex-determining region Y-box transcription factor 9; FOXL2: forkhead box L2 gene; DAX1: dosage-sensitive sex reversal, adrenal hypoplasia congenital critical region on the X chromosome, gene 1; SRY/TDF: sex-determining region Y protein/testis-determining factor gene Image credits - Ricardo Pasquini Neto, Maria Letícia Carnielli Tebet, Ohana Ivanski Dória de Vasconcelos, Mariana Faucz Munhoz da Cunha, and Maria Cristina Figueroa Magalhães

Duplication of dosage-sensitive sex reversal, adrenal hypoplasia congenital critical region on the X chromosome, gene 1 (DAX1) and WNT family member 4 gene (WNT4) and haploinsufficiency of sex-determining region Y-box transcription factor 9 (SOX9), steroidogenic factor-1 (SF1), Wilms tumor suppressor gene (WT1), and doublesex and mab-3 related transcription factor 1 (DMRT1) have also been described as being responsible for the development of 46,XY DSD [3,15]. One of the best understood pathophysiological mechanisms involves DAX1, which is in equilibrium with SRY/TDF. When the ratio becomes 2:1, the inactivation of SRY/TDF, anti-Müllerian hormone, and SOX9 occurs. This leads to the development of gonadal dysgenesis (Figure 4) [2,14].

Frasier et al., Denys et al., and Drash et al. described cases of chronic kidney disease in female patients with XY gonadal dysgenesis and WT1 gene mutations [16-18]. Frasier syndrome is a nephrotic syndrome caused by focal segmental glomerulosclerosis with a slow progression to renal failure. Denys-Drash syndrome affects children in their first years of life and is associated with a greater predisposition to Wilms tumors; its nephrotic syndrome occurs due to diffuse mesangial sclerosis [19,20]. Although the patient presented with nephropathy, and these two syndromes are important differential diagnoses, the nephrotic syndrome criteria was not met, making the presence of a WT1 mutation unlikely; however, it was not sequenced in the patient.

Malignancies with bilateral gonadal involvement occur in approximately 30% of patients with sexual developmental disorders. Because abnormal gonads lack normal physiological functions, contact with the intra-abdominal environment and intensive proliferation of undifferentiated cells may lead to genetic mutations that induce germ cell tumors (gonadoblastoma and dysgerminoma). Physiopathogenesis is questioned as to whether the gene responsible for 46,XY DSD itself is also carcinogenic or whether malformed tissues are more prone to develop neoplasms [21]. The prophylactic removal of dysgenetic gonads is recommended to prevent the development of malignancies [1,3]. Unfortunately, this was not possible in the present case because the tumor developed before the diagnosis of dysgenetic gonads.

Dysgerminoma is the most common type of ovarian germ cell cancer and primarily affects adolescents, young women, and individuals with gonadal dysgenesis. Abdominal pain and bloating are the main symptoms at diagnosis due to the rapid growth and expansion of the tumor. Histologically, tumors are composed of clusters and sheets of monotonous polygonal cells with distinct eosinophilic cytoplasm, distinct cell boundaries, square-edged nuclei with prominent nucleoli, and rapid mitotic activity [22]. The presence of calcifications is also a characteristic and may simulate the development of follicles on ultrasound, as in the present case. Rarely, dysgerminomas produce estrogen when they express steroidogenic enzymes (such as P450 aromatase) in the stroma. This may cause peripheral precocious puberty or the development of secondary sexual characteristics in patients with disorders of sex development [23].

In general, patients with gonadal dysgenesis do not develop secondary sexual characteristics because their gonads are involuted or their stroma is replaced by fibrous content. These characteristics may be due to peripheral androgenic conversion or intrinsic hormonal production by existing germ cell tumors. In the present case, peripheral androgen conversion was considered responsible for the development of secondary sexual characteristics, since no signs of tumor hormone production were identified, and the patient had central obesity. Patients with higher body mass index tend to have greater peripheral androgen conversion, once the amount of adipose tissue and aromatase enzymes is directly proportional to the amount of estrogen produced [24].

The psychological aspect of the patient was a consequence of a diagnosis of 46,XY DSD. Pediatricians, geneticists, endocrinologists, gynecologists, oncologists, psychiatrists, psychologists, nursing staff, and social assistants should be involved within the spheres of care. At the moment of transition to adulthood, it is extremely important to have an appropriate transfer of care involving the entire multidisciplinary team to receive the patient in a new center. Throughout the process, good communication with trained professionals and support from family members must be valued to minimize fear and anxiety [5].

## Conclusions

The 46,XY DSD has a non-specific clinical presentation. Its diagnosis, in a phenotypically normal girl with a 46,XY karyotype, is associated with primary amenorrhea of ovarian etiology. As the presence of Y chromosome material in DSD increases the risk of germ cell tumor development, patients should be advised to undergo prophylactic bilateral salpingo-oophorectomy. The management of the condition is complex, requiring the collaborative work of inter- and multidisciplinary teams that embrace the patient in his or her complete biopsychosocial and spiritual integrity.
